# Perplexities

**Published:** 1851-05

**Authors:** 


### ARTICLE II.

### PERPLEXITIES.

The following precious moriceau will give our readers some idea of the troubles and perplexities attending the publication of a Medical Journal. We are very happy to say that there are but few cases of this character. In fact most of our patrons lay us under deep obligations for the polite and flattering treatment we receive at their hands. There are a few, however, that think we should know when, and to where they move, without their being at any trouble to keep us informed.

When we send one number to a subscriber, by his request, he owes for the rest of the volume, as our terms are payment in advance; and if we send the rest of the volume as he has directed, we have complied with our part of the contract.

We do not discontinue, except at the close of a volume, and if it is stopped before the close by the subscriber's request, we charge for the whole year.

Some think they are only responsible for what they get under such circumstances, even though we may have been at the expense of sending them the Journal for years. The law holds differently; but when a man is (as the author of this letter, and most of those who are governed by such principles are) out-lawed, what can we do?

We can bear to be quietly or politely robbed of our dues, with christian fortitude and some degree of composure, but when insult is added to injury, we feel like using the editorial weapon of defence, and give to such as do it a notoriety they might not otherwise attain. In the present instance we in good faith sent the Journal one year to Dr. B. Z. Alexandre, of Beloit, Wis., when finding our subscriber had left, we discontinued it. His name was given our agent as a new subscriber, when in Oshkosh, about a year ago, without any allusion to his having subscribed at Beloit, and we sent it to him another year regularly; and when our collector sent his bill in, he received the following reply which we copy *verbatim et literatum*:

OSKOSH, Wis., March 21, 1851.

Dear Sir,

Your's of the 28 inst I am just in receipt of and in reply I would state to you that I have never received but 3 Nos of your Medical Journal you state that it was send to my adress in Beloit I would state to you that I have not resided in that Town since the early part of the fall of 1848 Therefore it was the duty of the P. Master. to write you that they were not taken from the office on the receipt of the 1th or 2d no. it is very foolish for you to expect me to pay for that which I never received the 3d No last published I am ready to pay for and I wish it discontinued If you should have the good luck to collect the $6 you have charged me with and which I have never received no value for *Please be so kind as to let me know how when and where and who pays the cost &c* You will find it an up hill business no doubt, as I ow nothing and own nothing but always pay my dues your attention to the above will as you wrote me save trouble and expense to you instead of me.

ALEXANDRE M D

